# The Slower, the Better: Wide Complex Tachycardia Triggered by Flecainide in an Elderly Female

**DOI:** 10.1155/2022/1409498

**Published:** 2022-10-15

**Authors:** Ebubechukwu Ezeh, Maddie Perdoncin, Morgan K. Moroi, Mohammad Amro, Mohammed Ruzieh, Paul I. Okhumale

**Affiliations:** ^1^Department of Internal Medicine, Marshall University, Huntington, WV, USA; ^2^Pennsylvania State University, State College, PA, USA; ^3^The Palestinian Ministry of Health and Higher Education, State of Palestine; ^4^University of Florida, Gainesville, FL, USA; ^5^Cedar Valley Cardiovascular Center, Waterloo, IA, USA

## Abstract

Class IC antiarrhythmics are generally considered a safe means of treating many common arrhythmias such as atrial fibrillation (a-fib), atrial flutter (a-flutter), and paroxysmal supraventricular tachycardia (PSVT). Essentially, flecainide works by binding and blocking sodium channels more effectively at higher heart rates. However, this class of drugs is known to exhibit use dependence which could predispose patients to the development of malignant arrhythmias during episodes of tachycardia. In this case, we present a patient who was being treated with flecainide for a-fib who ultimately developed a wide complex tachycardia after her metoprolol was held.

## 1. Introduction

Antiarrhythmic medications have been around for over 100 years and are commonly prescribed for conditions such as atrial fibrillation. As the burden of cardiac arrhythmias increases in the United States, these medications have quickly become one of the mainstays of therapy. The primary goal of treatment with these agents in a-fib is to achieve pharmacological cardioversion and limit the number of arrhythmic episodes as well as decrease the number of hospitalizations and mortality associated with atrial fibrillation [1].

The antiarrhythmic medications have historically been classified according to the Vaughan-Williams system, which groups them based on their mechanism of action [2]. Class I antiarrhythmic drugs are known to work by inhibiting cardiac sodium channels. While this group has had a significant impact on patient treatment, it does have significant proarrhythmic side effects. This is particularly pronounced with the class IC antiarrhythmics such as flecainide. Flecainide works in a use-dependent manner, essentially blocking sodium channels more potently at faster heart rates. When this property is not taken into consideration during treatment, malignant arrhythmias can occur [3]. We present the case of a 70-year-old woman treated with flecainide who developed a wide complex tachycardia after her beta blocker therapy was discontinued. This case was presented as an abstract at the 2022 American College of Cardiology conference in Washington, D.C. [4].

## 2. Clinical Presentation

A 70-year-old woman with paroxysmal a-fib status post direct current cardioversion (DCCV) a few weeks prior presented to the emergency department (ED) with generalized weakness and altered mental status. She had a history of hypertension. A day prior to arriving at the ED, the patient's husband noted that she appeared pale and weak. She was taken to her local pharmacy where her heart rate was found to be 40-45 beats per minute (bpm) and her blood pressure was normal. Her medications included metoprolol 12.5 milligram (mg) twice a day, flecainide 75 mg twice a day, and apixaban 5 mg twice a day. Then, her primary care physician was notified and recommended holding metoprolol and close observation. Her symptoms, however, persisted, and she eventually presented to the emergency department.

**Figure 1:**
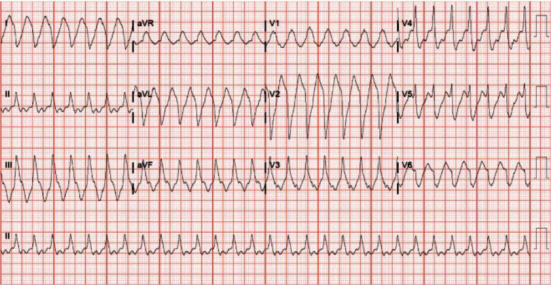
Presenting 12-lead electrocardiogram.

Upon initial evaluation, the patient was lethargic but responsive. She denied current feelings of weakness or altered mentation. Electrocardiogram (ECG) revealed wide complex tachycardia with a heart rate of 174 bpm and prolonged QRS of 270 ms (Figure 1), and cardiology was consulted. Serial ECGs continued to show wide complex tachycardia with heart rates between 180 and 190 bpm; the patient remained normotensive. Since the patient was stable, DCCV was deferred. Instead, 150 mg of amiodarone and 5 mg of metoprolol tartrate were given. Within a few minutes, the patient converted to normal sinus rhythm. She was then admitted to the hospital for observation. A transthoracic echocardiogram demonstrated newly depressed left ventricular systolic function.

The ECG revealed a regular wide complex tachycardia at a heart rate of 170 bpm and a prolonged QRS with duration of 270 ms (Figure 1). The following differential diagnosis was entertained including atrial flutter with 1 : 1 atrioventricular (AV) conduction, SVT with aberrancy, QRS widening attributable to flecainide, and ventricular tachycardia. Given the patient's exposure to flecainide and the absence of A-V dissociation (best appreciated in leads II, V3, and V4), atrial tachycardia or atrial flutter with 1 : 1 conduction was the topmost on the differential.

**Figure 2:**
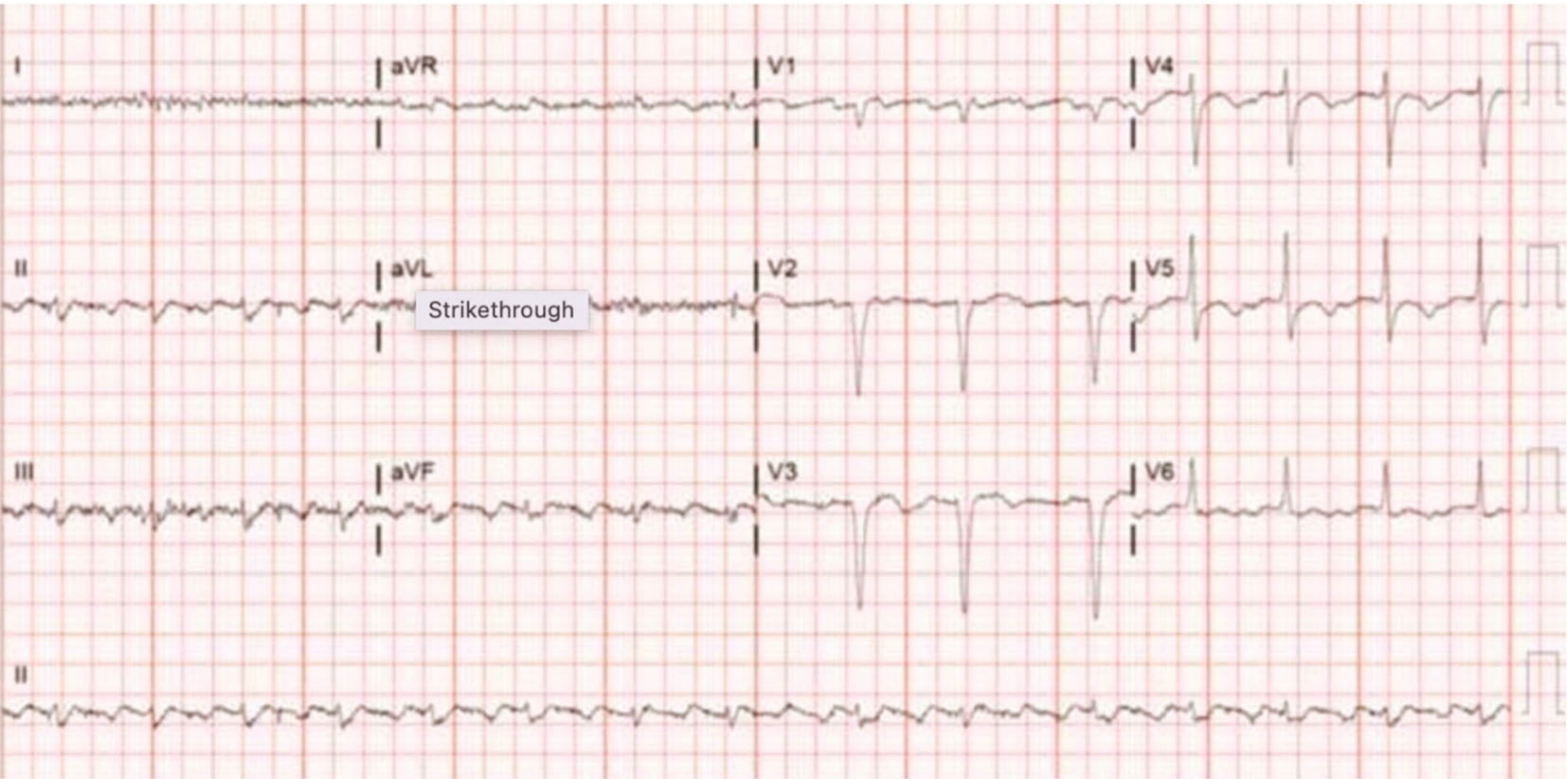
ECG showing atrial flutter after administration of metoprolol and amiodarone.

**Figure 3:**
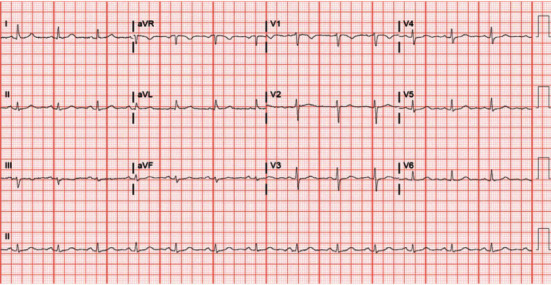
ECG demonstrating sinus rhythm.

The administration of amiodarone and metoprolol slowed the ventricular rate revealing the flutter waves as shown in Figure 2. Shortly after, the patient converted to sinus rhythm (Figure 3), and flecainide was stopped. The patient was maintained on continuous IV amiodarone (0.5 mg/min). She was subsequently switched to amiodarone 200 mg twice a day for 10 days followed by 200 mg daily afterwards. Metoprolol tartrate was increased to 50 mg daily. She also underwent an electrophysiology study, which confirmed typical atrial flutter, and had successful cavo-tricuspid-isthmus (CTI) ablation. Given her newly depressed left ventricular function, flecainide was not restarted. She was discharged home on amiodarone and metoprolol without recurrence of arrhythmias on follow-up. The metoprolol dose was halved to 25 mg upon discharge. The patient was also treated for urinary tract infection which was believed to have contributed to her initial weakness.

## 3. Discussion

Flecainide is a class IC drug that binds open-state sodium channels to increase the effective refractory period and slow conductance in atrial and ventricular tissues. It demonstrates the slowest unbinding kinetics of the class I antiarrhythmic drugs and exhibits use dependence, which captures how the degree of sodium channel blockade increases with increases in heart rate. Essentially, this class of antiarrhythmics is more potent at faster heart rates [5]. Flecainide also blocks the rapid components of the delayed rectifier current and reduces spontaneous calcium release by the sarcoplasmic reticulum through ryanodine receptor inhibition [6].

In terms of noticeable ECG changes, the effect of flecainide is greatly enhanced with higher heart rates and can result in significant intraventricular conduction delays, causing a widened QRS complex [4, 7]. Flecainide can also organize atrial fibrillation into slow atrial tachycardia or atrial flutter due to decreased atrial tissue conductance. While typical atrial flutter usually occurs at rates up to 300 bpm, the atrial rate can be much slower in the presence of flecainide, as seen in our patient. Furthermore, without adequate use of a concomitant AV nodal blocking agent, this slow flutter can be conducted in a 1 : 1 ratio, resulting in a rapid ventricular response [8–10]. Our patient's newly depressed left ventricular function was most likely a result of atrial flutter [11]. Additionally, our patient had a history of hypertension, which could also be a potential etiology of her ventricular dysfunction.

When treating a patient with flecainide, it is important to monitor QRS duration. An increase in QRS duration greater than 15-20% puts the patient at risk for development of arrhythmias and indicates that the dose may need to be lowered [5]. It may also be beneficial to do an exercise stress test prior to starting a patient on flecainide to ensure that elevated heart rates during everyday activities will not significantly prolong the QRS and predispose them to the development of arrhythmias. Additionally, in patients with pacemakers, flecainide increases the pacing threshold [5]. If the pacing is not appropriately adjusted, it may lead to dangerous arrhythmias. Despite these concerns, in general, flecainide assumes a low risk of causing malignant arrhythmias in a structurally normal heart.

## 4. Conclusion

This case demonstrates the use-dependent properties of the class IC antiarrhythmic medication flecainide. It is important to note that at higher heart rates, flecainide can induce a dangerous arrhythmia by interacting with its target sodium channel more potently. In this case, an elevated heart rate induced by cessation of beta blocker therapy resulted in the development of a wide complex tachycardia.
